# Innate and Adaptive Immunity in Long-Term Non-Progression in HIV Disease

**DOI:** 10.3389/fimmu.2013.00095

**Published:** 2013-04-24

**Authors:** John Zaunders, David van Bockel

**Affiliations:** ^1^Centre for Applied Medical Research, St Vincent’s HospitalDarlinghurst, NSW, Australia; ^2^Kirby Institute, University of New South WalesSydney, NSW, Australia

**Keywords:** HIV-1, long-term non-progressors, elite controllers, cytotoxic T lymphocytes, cell-mediated immunity

## Abstract

Long-term non-progressors (LTNP) were identified after 10–15 years of the epidemic, and have been the subject of intense investigation ever since. In a small minority of cases, infection with nef/3′LTR deleted attenuated viral strains allowed control over viral replication. A common feature of LTNP is the readily detected proliferation of CD4 T-cells *in vitro*, in response to p24. In some cases, the responding CD4 T-cells have cytotoxic effector function and may target conserved p24 epitopes, similar to the CD8 T-cells described below. LTNP may also carry much lower HIV DNA burden in key CD4 subsets, presumably resulting from lower viral replication during primary infection. Some studies, but not others, suggest that LTNP have CD4 T-cells that are relatively resistant to HIV infection *in vitro*. One possible mechanism may involve up-regulation of the cell cycle regulator p21/waf in CD4 T-cells from LTNP. Delayed progression in Caucasian LTNP is also partly associated with heterozygosity of the Δ32 CCR5 allele, probably through decreased expression of CCR5 co-receptor on CD4 T-cells. However, in approximately half of Caucasian LTNP, two host genotypes, namely HLA-B57 and HLA-B27, are associated with viral control. Immunodominant CD8 T-cells from these individuals target epitopes in p24 that are highly conserved, and escape mutations have significant fitness costs to the virus. Furthermore, recent studies have suggested that these CD8 T-cells from LTNP, but not from HLA-B27 or HLA-B57 progressors, can cross-react with intermediate escape mutations, preventing full escape via compensatory mutations. Humoral immunity appears to play little part in LTNP subjects, since broadly neutralizing antibodies are rare, even amongst slow progressors. Recent genome-wide comparisons between LTNP and progressors have confirmed the HLA-B57, HLA-B27, and delta32 CCR5 allelic associations, plus indicated a role for HLA-C/KIR interactions, but have not revealed any new genotypes so far. Nevertheless, it is hoped that studying the mechanisms of intracellular restriction factors, such as the recently identified SAMHD1, will lead to a better understanding of non-progression.

## Introduction

Prior to the introduction of potent combination antiretroviral therapy (cART), in developed countries, the median time from seroconversion to AIDS-defining illness was 8–10 years (reviewed in Pantaleo and Fauci, 1996). However, slowly patients were recognized who showed little or no clinical progression (Lifson et al., 1991; Learmont et al., 1992), later termed long-term non-progressors (LTNP) (Pantaleo and Fauci, 1996). Recent data from prospective cohorts suggest that only a very small minority of untreated patients are LTNP and even fewer are able to spontaneously control HIV-1 replication, with undetectable plasma viral loads and very stable CD4 cell counts (Madec et al., 2005; Goujard et al., 2009; Okulicz et al., 2009; Mandalia et al., 2012); such patients are commonly referred to as Elite Controllers (EC) (Blankson et al., 2007; Walker, 2007).

## Viral Fitness in LTNP and EC

One of the earliest studies in non-progression was identification of the Sydney Blood Bank Cohort (SBBC), where all subjects were infected via blood transfusions from a single donor, who was an LTNP himself (Learmont et al., 1992). It was found by full HIV-1 genome sequencing that the SBBC patients had been infected by a highly attenuated nef/LTR deleted mutant strain (Deacon et al., 1995). However, extensive analyses of viral isolates from other cohorts of EC have found that such patients are rarely infected with similar nef-deleted attenuated strains of HIV-1 (Miura et al., 2008; Pushker et al., 2010). Instead, many LTNP and EC can have fully replication competent HIV-1 (Alexander et al., 2000; Blankson et al., 2007; Lamine et al., 2007), although, more detailed studies suggest that there can be subtle decreases in function of Nef (Mwimanzi et al., 2013) or rare polymorphisms and reduced function in other genes (Alexander et al., 2000; Miura et al., 2010). In addition, env genes from EC subjects appear to be subtly different to chronic progressors, with reduced rates of entry into cells at defined levels of cell surface CD4 and CCR5 expression (Lassen et al., 2009). In some cases, transmitted drug resistant HIV-1 may have an associated fitness cost and result in lower viral load in the recipient (Harrison et al., 2010).

Therefore, there is evidence that in some LTNP and EC individuals, infection with HIV-1 exhibiting low viral replicative fitness may help explain viral control. However, it is also possible that in many LTNP and EC, other immunological factors such as pressure exerted by their CD8 cytotoxic T lymphocytes (CTL) upon viral sequences vital to replication, has led to mutations that reduce viral fitness (reviewed in Goulder and Watkins, 2004). Studies of experimental SIV infection (Allen et al., 2000; O’Connor et al., 2002) and recent studies of primary HIV-1 infection (PHI) demonstrate that CTL escape mutations occur very early after infection (Goonetilleke et al., 2009; Miura et al., 2010; Henn et al., 2012). In retrospective studies of LTNP and EC who are defined after many years of infection, it may be difficult to determine which came first, infection with low replicative fitness HIV-1, or immune pressure leading to unfit escape mutants, as discussed in detail below.

A proportion of subjects who are initially viral controllers after PHI exhibit viral breakthrough, suggesting that many factors are involved in prolonged EC status (Madec et al., 2005; Goujard et al., 2009; Okulicz et al., 2009). In the case of the SBBC subjects, three out of four who had low but detectable viral loads, eventually had further mutations that resulted in less attenuated HIV-1 and disease progression after 14–18 years (Gorry et al., 2007; Zaunders et al., 2011). Similarly, HLA-B27 LTNP with low viral loads can exhibit step-wise mutations leading to compensation and a relatively high replication escape mutant strain of HIV-1 (Kelleher et al., 2001) associated with clinical progression (Goulder et al., 1997; Ammaranond et al., 2011).

## Viral Load in LTNP and EC

Following the introduction of plasma HIV-1 RNA viral load measurement by PCR, it was found that HIV+ patients exhibited a very broad spectrum of viral load, from low to high levels, over six orders of magnitude, and that a longer time to AIDS-defining illness was highly correlated with lower viral load (Mellors et al., 1997). LTNP have significantly lower plasma HIV RNA viral loads than progressors (Panteleo et al., 1995), and by definition EC subjects have undetectable plasma viral load. The level of HIV RNA plasma is believed to reflect ongoing high levels of viral replication, particularly in lymphoid tissues, since virions in plasma are reportedly produced and cleared very rapidly, with a half-life of the order of 0.5–2 h (Ramratnam et al., 1999). Therefore, LTNP and EC have greatly reduced viral replication in tissues.

In addition to HIV RNA in plasma, the levels of HIV DNA in PBMC have also been measured by quantitative PCR, and in general the two parameters are highly correlated (Rouzioux et al., 2005), although the observed range of HIV DNA levels only encompasses three orders of magnitude. Again, a lower level of HIV DNA in PBMC is correlated with delayed clinical progression to AIDS (Rouzioux et al., 2005). Accordingly, LTNP and EC have been found to have significantly lower levels of HIV DNA in PBMC, compared to other patients (Panteleo et al., 1995; Lambotte et al., 2005; Dalmasso et al., 2008; Julg et al., 2010a; Graf et al., 2011; Zaunders et al., 2011; Mendoza et al., 2012a). This low level of HIV DNA is consistent with early studies showing that it was relatively difficult to isolate HIV-1 from plasma or PBMC from such patients, for example in the SBBC (Learmont et al., 1992; Deacon et al., 1995), and also in later studies using highly sensitive co-culture methods (Blankson et al., 2007).

Since HIV-1 dissemination throughout the lymphoid system occurs shortly after infection, the question arises whether it is critical events during PHI that determine the reduced viral burden in LTNP/EC. These considerations indicate that the formation of the reservoir of HIV DNA within infected cells is different for LTNP and EC subjects during PHI, resulting in overall reduced viral burden.

Viral replication is at its highest during the very early stages of PHI (Clark et al., 1991), then decreases to an apparent long-term equilibrium, that is referred to as the set point (Mellors et al., 1995). The peak of viral load, the subsequent kinetics of the decrease, and the set point are all quite different for each individual patient (Kaufmann et al., 1998), strongly suggesting that LTNP or EC status could be established at this time. During this period of high viral load, a considerable number of memory CD4+ T-cells are infected with proviral HIV DNA that integrates into genomic DNA, to form the cellular basis of the HIV-1 reservoir that is resistant to cART (Chun et al., 1998). The reservoir formed during PHI may possibly be slightly less long-lived (Chun et al., 2007) and slightly smaller than the reservoir established by the time of 1 year of chronic infection (Strain et al., 2005; Koelsch et al., 2011).

Primary HIV-1 infection is a period when a vigorous immune response to HIV-1 antigens occurs, with appearance of antibodies (Cooper et al., 1985), as well as a dramatic increase in activated CD4 and CD8 T-cells (Zaunders et al., 1995). It is generally believed, from experimental SIV infection in Rhesus macaques, that the initial focus of infection occurs in resting CD4 T-cells in genital mucosa, followed very shortly by spread to lymphatic tissues (Haase, 2010). Dissemination and formation of the reservoir is believed to be fueled by inflammation and infection of responding activated CD4 T-cells (Haase, 2010), and it has been proposed that some of these activated cells revert to long-lived resting memory T-cells containing replication competent provirus (Han et al., 2007). We have found that much of the HIV DNA in PBMC during the most acute stage of PHI was already found in long-lived resting CD4 memory T-cells that expressed the IL-7 receptor (Zaunders et al., 2006b), rather than the highly activated short-term effector CD4 T-cells that peak during PHI (Zaunders et al., 2005). At the peak of PHI, it is unlikely that activated cells have already reverted to resting cells. Also, it is widely believed that most HIV-specific CD4 T-cells, which should represent the bulk of activated CD4 T-cells during PHI, are lost, presumably due to infection, unless patients commence cART (Rosenberg et al., 1997), consistent with the report that HIV-specific CD4 T-cells are preferentially infected with HIV DNA (Douek et al., 2002).

Altogether, the very low levels of HIV-specific CD4 T-cells after PHI suggest that it is unlikely that they represent the majority of the long-lived resting memory CD4 T-cell reservoir for proviral HIV, as suggested by the infected activated cells reverting to resting cells model. Rather, direct infection of long-lived resting IL-7R+ CD4 T-cells, while they transit through infected lymphoid tissue, may be more likely to result in the cART-resistant reservoir, and that IL-7 maintains homeostasis of these memory CD4 T-cells (Chomont et al., 2009). The antigen specificity of the HIV DNA-infected memory CD4 T-cell reservoir is unclear at present.

The issue becomes whether PHI is different for LTNP or EC, and whether their outcome is lower HIV RNA and HIV DNA viral load due to reduced replication and dissemination, compared to other patients. Approximately half of EC are HLA-B57 (Migueles et al., 2000) (see below) and an early report concluded that HLA-B57 individuals presented significantly less often with symptomatic PHI, suggestive of a milder course of disease (Altfeld et al., 2003). Furthermore, six out nine of the HLA-B57 PHI subjects in that study had relatively low plasma viral loads (Altfeld et al., 2003). However, in another prospective study of PHI, eight EC were identified amongst 211 untreated PHI subjects (out of a total of 964 subjects enrolled), and there was no significant difference in symptomatic PHI (Goujard et al., 2009), but there was a clearly significant difference in HIV RNA viral load. Similarly, we found that patients who exhibited better control of HIV RNA, during treatment interruption after 6–12 months cART commenced during PHI, had a significantly lower HIV RNA level at baseline, by multivariate analysis (Bloch et al., 2006), and this correlated with lower levels of integrated HIV DNA prior to cART (Lewin et al., 2008). Another large natural history study showed that viral control was established in the majority of EC within 1 year of seroconversion, but characteristics of PHI were not reported for this cohort (Okulicz et al., 2009). However, within this same group of patients, lower HIV DNA levels in purified CD4 T-cells at baseline correlated with longer time to clinical progression (Ganesan et al., 2010).

Altogether, there is consistent evidence indicating that LTNP/EC emerge from PHI with low levels of chronic HIV-1 replication, and possibly low levels of long-lived memory CD4 T-cells infected with HIV-1 DNA, but the rarity of these patients to study during PHI makes it difficult to power studies for greater statistical significance.

## Are CD4 T-Cells from LTNP and EC Resistant to HIV-1 Infection?

If LTNP and EC have lower peaks of viral load during PHI and lower viral set points after PHI, the possibility arises that their CD4 T-cells are simply more difficult to productively infect with HIV-1.

At the time of the discovery that CCR5 was the major co-receptor for HIV-1 on the surface of CD4 T-cells, studies showed that subjects heterozygous for the CCR5 Δ32 mutation had lower plasma viral loads, slower declines in CD4 T-cell counts, and delayed progression (Dean et al., 1996; Huang et al., 1996). An increased prevalence of heterozygosity for the CCR5 Δ32 mutation was found in some well-defined cohorts of LTNP (Morawetz et al., 1997; Stewart et al., 1997), but not all, although a meta-analysis of many cohorts confirmed that this genotype had a strong protective effect delaying progression to AIDS-defining illness (Ioannidis et al., 2001). This heterozygosity was believed to reduce cell surface CCR5 by a dominant negative effect of the Δ32 mutant allele (Benkirane et al., 1997), presumably limiting HIV-1 entry, although the effect on cell surface CCR5 is quite variable for individual Δ32 heterozygous subjects (de Roda Husman et al., 1999). The prevalence of Δ32 heterozygosity in EC appears be statistically higher than in the general population (Pereyra et al., 2010), but not dramatically so, and where reported, the large majority of EC are wild-type for the CCR5 allele (Lambotte et al., 2005; Pereyra et al., 2008).

Since Env from EC isolates required more CCR5 and CD4 for entry than env from chronic progressors (Lassen et al., 2009), this suggested the possibility that subtle changes in both env and CCR5 expression in the same patient could together contribute significantly to viral control. Also, the Δ32 mutation, plus two other previously described SNP’s in the CCR5-CCR2 locus, were the only polymorphisms other than HLA alleles that were significant in a genome-wide genetic association study of viral controllers, compared to progressors (Pereyra et al., 2010).

Direct study of the ability of HIV-1 to infect CD4 T-cells from EC has yielded conflicting results. Most reports have found that CD4 T-cells from EC can be infected with autologous or laboratory strains of HIV-1, using activated CD4 T-cells (Wang et al., 2002; Blankson et al., 2007; Lamine et al., 2007; Julg et al., 2010a) or resting CD4 T-cells (O’Connell et al., 2011; Rabi et al., 2011). However, one study recently reported that CD4 T-cells from EC were relatively resistant to HIV infection *in vitro*, describing a role for the tumor suppressor, p21Cip1/Waf1, although a mechanism for the effect of reduced reverse transcription was unclear (Chen et al., 2011). A second report found that macrophages from EC have low susceptibility to HIV-1 infection, while CD4 T-cells from the same patients, had reduced reverse transcription in the first round of infection, similar to the first report, although no direct role for p21 could be found (Saez-Cirion et al., 2011).

Interestingly, CD4 T-cells in PBMC from EC had very low levels of integrated HIV DNA, but relatively high levels of episomal 2-LTR circles (Graf et al., 2011). The authors did not find a block to integration, but rather concluded that in these patients, CD8 CTL pressure possibly rapidly removed infected cells, leaving a proportionately higher population of non-productively infected cells (Graf et al., 2011).

A potential problem with directly comparing CD4 T-cells from EC to CD4 T-cells from progressors is the effect of chronic HIV-1 infection associated with increased rates of activation. We originally found that EC subjects in the SBBC had much lower levels of activation (within the normal range), and greatly increased numbers of resting memory CD4 T-cells, compared to progressors (Zaunders et al., 1999b). This was reported again in a later study of EC (Potter et al., 2007), and has since been also suggested in another study of EC, although not explicitly reported (Hunt et al., 2008). In this latter study, activation was barely elevated, particularly in EC with CD4 cell counts ≥600 cells/mm^3^, compared to HIV-uninfected controls (Hunt et al., 2008), meaning that EC had greatly increased resting cells compared to progressors.

The HIV-1 restriction factor SAMHD1 is an interferon inducible enzyme in myeloid cells (Goldstone et al., 2011; Hrecka et al., 2011) which limits HIV-1 infection through depleting available dNTP for reverse transcription (Lahouassa et al., 2012). Identified using mass-spectrometry as a counteracted target for the HIV-2/SIV protein Vpx (Laguette et al., 2011), SAMHD1 highlights the role of intrinsic host factors in limiting reservoir generation particularly in resting CD4 T cells. Polymorphisms in the SAMHD1 gene currently demonstrate no strong associations with non-progressive status (Coon et al., 2012), however it is clear that the role of SAMHD1 as an intrinsic factor associated with EC has not been thoroughly investigated.

Another restriction factor, TRIM5a, has been studied for potential polymorphisms in LTNP, although this restriction factor is much more active against SIV in human cells than it is against HIV-1 (Nakayama and Shioda, 2012). One study reported that a H43Y mutation in TRIM5a was associated with accelerated progression (van Manen et al., 2008), although this was not confirmed in other studies (Nakayama and Shioda, 2012).

Altogether there is not clear-cut evidence that EC have CD4 T-cells that are resistant to HIV-1 infection. Direct comparisons of PBMC from LTNP/EC versus progressors must be critically assessed to take into account possible differences in the relative proportions of resting and activated CD4 T-cells, since resting cells are known to be harder to infect *in vitro* (Zack et al., 1990).

## Do LTNP and EC have a More Vigorous Immune Response to HIV-1 Antigens during PHI?

An effective CD8 T-cell response is believed to be important in reducing the peak of viral load after PHI (McMichael et al., 2010), and the association of such responses with LTNP and EC status is discussed below.

However, there is also a significant CD4 T-cell response to HIV infection as well during PHI (Rosenberg et al., 2000; Oxenius et al., 2001; Gloster et al., 2004; Kaufmann et al., 2004; Zaunders et al., 2005; Maenetje et al., 2010; Riou et al., 2012). We have consistently found that there is a transient, greatly increased rate of activation and proliferation of CD4 T-cells which results in high levels of CD38high, CCR5+, Ki67+, and CD127low cells (Zaunders et al., 1995, 2001, 2005), making ideal targets for highly productive HIV-1 infection. A similar population of activated CD4 T-cells is observed from day 10 to day 14 following vaccinia inoculation in healthy adult volunteers (Zaunders et al., 2006a). It may be counter-intuitive, but a relatively restrained CD4 T-cell response could be beneficial if fewer activated cells are generated during PHI. Rather than resistance of CD4 T-cells to infection, a reduced immune response and lower number of target cells generated for productive infection during PHI may play an important part in LTNP/EC status. Consistent with this possibility, a recent study of a large natural history cohort showed that a lower proportion of such activated CD4 T-cells at baseline was highly correlated with lower cell associated HIV DNA levels (Ganesan et al., 2010), lower plasma viral loads (Okulicz et al., 2009), and better long-term outcome (Ganesan et al., 2010).

The role of an anti-HIV-1 CD4 T-cell response in simultaneously controlling HIV-1 replication, but at the same time contributing target cells, is obviously complex.

There are numerous reports of the deleterious effect of high HIV-1 viral replication in patients on their CD4 T-cell function *in vitro* and *in vivo*, including: (i) reduced IL-2 production (Clerici et al., 1989), and we have found recently this is associated with over-expression of the transcriptional repressor Blimp-1 in CD4 T-cells from progressors, compared to LTNP (Seddiki et al., 2012); (ii) reduced proliferation in response to standard mitogens and antigens (Miedema et al., 1988; Clerici et al., 1989), including response to HIV antigens (McNeil et al., 2001), probably secondary to reduced IL-2 production; (iii) increased CTLA-4 expression, which is a known negative regulator of CD4 T function (Zaunders et al., 2006b; Kaufmann et al., 2007); (iv) increased PD-1 expression (Day et al., 2006), although the exact mechanism of negative function is not defined, but may be linked to increased IL-10 production (Brockman et al., 2009; Said et al., 2010) and the transcription factor BATF (Quigley et al., 2010); (v) fibrosis of lymph nodes, possibly leading to reduced availability of IL-7 mediated survival signals (Estes et al., 2008), combined with reduced proportions of IL-7R+ CD4 T-cells possibly due both to activation and to reduced homeostasis (Sasson et al., 2006). It is very important to note, however, that treating patients with subcutaneous IL-2 in a series of very large clinical trials did not lead to any clinical benefit (Abrams et al., 2009).

In contrast, LTNP and EC subjects are best distinguished by the observation that CD4 T-cells in their PBMC proliferate *in vitro* in response to HIV-1 antigens, described in numerous reports (reviewed in detail in Dyer et al., 2008). These results strongly suggest that LTNP and EC subjects have a significant population of circulating HIV-specific memory CD4 T-cells. Furthermore, CD4 T-cells from EC subjects responded to, on average, 10-fold lower concentrations of Gag peptides *in vitro* than corresponding cells in other HIV+ subjects, suggesting that the HIV-specific CD4 T-cells from EC had much higher avidity TCR (Vingert et al., 2010).

These CD4 T-cells may have an indirect effect by helping a potent CD8 response to control HIV replication (Kalams and Walker, 1998), and while it is still unclear how this may occur, production of IL-2 has been implicated (Lichterfeld et al., 2004) and more recently IL-21 (Yue et al., 2010; Chevalier et al., 2011; Williams et al., 2011), although improved CD8 function may be also maintained by autochthonous production of IL-2 (Zimmerli et al., 2005) or IL-21 (Williams et al., 2011).

**Figure 1:**
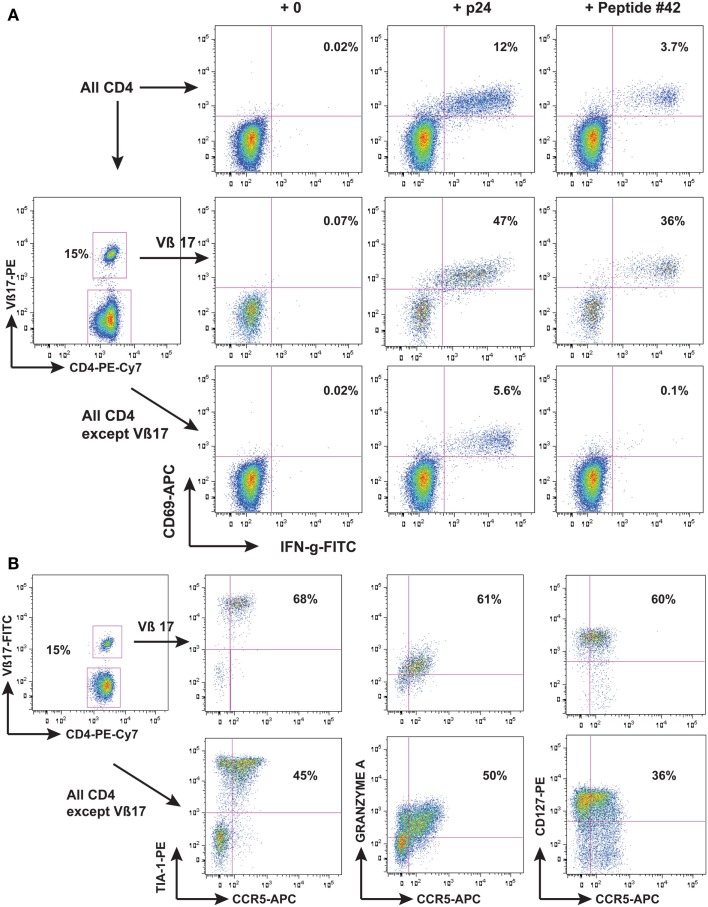
**LTNP 100149 CD4 response to HIV-1 p24**. Flow cytometry histograms showing that **(A)** subject LTNP 100149 cells had a very large CD4 response [**(A)**, upper row of histograms] to HIV p24 (baculovirus recombinant) and also to Gag 15-mer peptide SPEVIPMFSALSEGA (peptide #42 from NIH reagents program). LTNP 100149 had an expansion of TCR Vβ17 CD4 T cells [**(A)**, middle row] that had half the original response to p24 and all the original response to peptide #42, while CD4 T cells excluding TCR Vβ17 cells [**(A)**, lower row] had half the original response to p24, but no response to peptide #42. **(B)** Immunophenotyping of LTNP100149 TCR Vβ17+ CD4 T cells [**(B)**, upper row] showed that they had very high expression of the cytotoxic granule marker TIA-1, dim CCR5, Granzyme A, and CD127 (IL-7Rα chain), relative to CD4 T cells excluding TCR Vβ17 cells [**(B)**, lower row].

Another possibility however, is a direct anti-viral effector function of HIV-specific CD4 T-cells. We found that one individual LTNP, who had an extremely low rate of HIV-1 replication (Wang et al., 2002), had a very vigorous CD4 proliferation in response to HIV-1 Gag, and when these cells were identified as an expansion of TCR Vβ17+ cells, it allowed detailed study of their phenotype to be performed (Zaunders et al., 2004). It was found that these Vβ17+ CD4+ T-cells had a cytotoxic phenotype (shown in Figure 1) and were able to lyse autologous B cells coated with the cognate peptide (Zaunders et al., 2004). Another study in parallel similarly found that an LTNP with a very large proliferative CD4 response also had cytotoxic CD4 T-cells specific for a Gag peptide (Norris et al., 2004). In both cases, the epitope overlapped with a CD8 immunodominant epitope recognized by CD8 CTL from HLA-B57 LTNP and EC (see below). Furthermore, the same epitope was significantly more commonly recognized by CD4 T-cells from EC subjects, and resulted in greater proliferation *in vitro*, than for CD4 T-cells from other HIV+ subjects (Vingert et al., 2010).

We had previously found that HIV-1 infection was associated with an increase in circulating CD4+ T-cells that contained perforin (Appay et al., 2002). These perforin+ CD4 T-cells could be seen even as early as during PHI (Appay et al., 2002) and we subsequently found that HIV-specific CD4 T-cells during PHI highly expressed markers of CTL (Zaunders et al., 2005). These results suggest that CD4 effector cells with a CTL phenotype are a surprisingly common component of an anti-viral response, which we confirmed by detailed study of the primary immune response during the first 3 weeks after vaccinia inoculation in healthy adults (Zaunders et al., 2006a). Similarly, in many individuals, CMV-specific CD4 T-cells can have a cytotoxic phenotype (Gamadia et al., 2004; Zaunders et al., 2004; Casazza et al., 2006), including during primary CMV infection (Gamadia et al., 2004). The results described above suggest that they can contribute significantly to viral control in rare HIV+ patients. A recent report of 11 subjects studied during PHI suggested that a higher level of Granzyme A+ HIV-specific CD4 T-cells during PHI correlated with a slower rate of progression and better clinical outcome (Soghoian et al., 2012). Evidence from SIV infected EC monkeys has also suggested that there are CD4 CTL that control SIV replication *in vivo* (Sacha et al., 2009; Burwitz et al., 2012). The role of CD4 CTL has not been studied to same extent as CD8 CTL, but is gaining greater appreciation in other viral infections (Sant and McMichael, 2012).

Interestingly, another study has reported that lower viral loads in LTNP were associated with CD4 responses skewed toward Gag epitopes, while progressor subjects had responses skewed toward Env epitopes (Ranasinghe et al., 2012). This is highly reminiscent of an earlier corresponding result in CD8 responses (Kiepiela et al., 2007), discussed below. HLA-DRB1*13 presents another epitope that is from a highly conserved section of HIV Gag, and it was found that subjects treated during PHI who were HLA-DRB1*13 and had responses to this epitope, had a better clinical response to therapy (Malhotra et al., 2001). Another study has confirmed that subjects with HLA-DRB1*13 did better in HIV clade C infection (Julg et al., 2011).

A possible explanation to reconcile all these observations may be that LTNP have reduced CCR5 expression on a critical CD4 effector subset, especially during PHI, making them less susceptible to infection and loss. Central memory CD4 T-cells (Tcm) are believed to be a long-lived subset of CD4+ T-cells and are more proliferative than effector memory CD4 T-cells (Tem) (Sallusto et al., 2004). In a study of HLA-B27 and HLA-B57 LTNP, Tcm contained particularly low levels of HIV DNA, which was associated with lower levels of cell surface CCR5 expression on Tcm (Descours et al., 2012). Consistent with these results, another study has very recently been presented at CROI 2013, that found that EC had lower levels of CCR5 on activated Tcm (Ramirez Kitchen et al., 2013).

Altogether, there is not clear-cut evidence that EC have CD4 T-cells that are resistant to HIV-1 infection. Rather than EC having a greater magnitude immune response to HIV-1, they may have a less broad response that is more focused on critical Gag epitopes, which may include cytotoxic CD4 T-cells and in which activated HIV-specific CD4 Tcm cells may have lower levels of CCR5 expression. Conversely, progressors with higher viral loads, associated with continual generation of diverse quasispecies in Env sequences, may maintain higher levels of activated CCR5+ CD4 T-cells, beginning during PHI (Zaunders et al., 2001, 2005) and continuing during chronic infection. If these cells tend to respond vigorously to Env epitopes, this may increase target cell numbers and contribute to continuing higher viral load, more dysregulated CD4 homeostasis and greater decline in the number of resting CD4 T-cells.

## HLA Associations with HLA-B57 and HLA-B27, and CD8 T-Cell Control of Viral Replication in LTNP and EC

Patients classified as non-progressors are overrepresented by individuals with alleles from the HLA-Bw4 serotype, namely HLA-B*2705, which is overrepresented in Caucasian LTNP cohorts and HLA-B*5701 and HLA-B*5703, from Caucasian and African cohorts, respectively (Carrington and O’Brien, 2003). The multinational HIV Controllers Study completed a genome-wide association study to determine any association of immunogenetics with viral control across multiple ethnicities (Pereyra et al., 2010). While >300 SNP associations were identified (all within the MHC complex), non-progression for European individuals exclusively fell in order of an odds-ratio for delayed progression within the HLA alleles; HLA-B*5701/B*5201, HLA-B*2705, and HLA-B*1402 (Pereyra et al., 2010). In particular, roughly half of EC are HLA-B57 as originally reported by Migueles et al. (2000).

These alleles are capable of mounting effector CD8+ T-cell responses that target several parts of HIV-1 (Goulder et al., 1996, 1997, 2001a). In the majority of cases however, these patients mount a narrowed but highly effective immunodominant response to the p24 capsid protein of HIV Gag.

The capsid protein consists of two helical domains; the C-terminal domain (residues 148–231) which is critical for Gag dimerization and is highly conserved among retroviruses. The N-terminal domain (residues 1–146) functions in maturation of the virus and correct viral disassembly following viral entry. The HLA-B*2705 target epitope KRWIILGLNK (KK10) lies along helix 7, while importantly, the B*5701 epitopes KAFSPEVIMF (KF11) and TSTLQEQIGW (TW10) lie along the respective helices 2 and 6.

Targeting of these epitopes, which are critical to viral function, strongly suggests the active selection of an attenuated virus in order to escape from CD8+ T-cell surveillance. Mutations at these epitopes are a *casus fortuitus*, an “unavoidable accident” of nature that are required to allow immune escape, but come at a high price, resulting in significant loss of fitness (Goulder and Watkins, 2004).

The definitive example of viral immune escape occurs in individuals expressing HLA-B*2705, in which a series of concurrent mutations takes place during the course of infection, in the KK10 epitope. An initial mutation at position 6 from Leu to Met (L268M) early in infection (Lichterfeld et al., 2007), is considered an attempt to escape T-cell receptor recognition. Studies suggest an antagonistic effect from this change (Klenerman et al., 1994), despite no difference observed in peptide binding affinity to the MHC class I molecule (Ammaranond et al., 2003). This mutation is tightly clustered with a second Arg (R) to Lys (K) mutation at position 2 (R264K) (Kelleher et al., 2001), which is a prerequisite residue for binding to the HLA-B*2705 molecule. The R264K mutation is always observed with a simultaneous upstream compensatory Serine to Alanine (S173A) mutation, required for independence of host cyclophilin-A for post-entry uncoating (Schneidewind et al., 2007). MHC binding affinity is then significantly reduced and capacity for presentation of this epitope is lost. Importantly, the timing of the R264 mutation is commonly observed no earlier than 8–10 years following infection, demonstrating the critical importance of viral fitness to clinical outcome.

Perinatal transmission of an existing R264K mutation results in the incapacity to mount an equivalent immunodominant response (MacDonald et al., 1998; Goulder et al., 2001b); however mechanistically, this differs from transmission of the T242N TCR escape mutation generated from the immunodominant HLA-B*5701 (TW10) epitope (Schneidewind et al., 2009; Miura et al., 2010). In a dissimilar fashion to R264K, haplo-identical infants are able to maintain fitness-attenuated virus through p24 Gag (Goulder et al., 2001b). The impact of HLA-B*5701 is thus apparent from the onset of infection and, among other factors, may contribute to a reduced viral reservoir. Ultimately, it is a combination of viral and host factors which contribute to disease progression in these individuals (Norstrom et al., 2012).

## CD8 Effector Function in LTNP and EC

The classic CD8 effector function is cytotoxic activity toward infected target cells. Knockout of perforin in murine models yields a very clear phenotype in which control over non-cytopathic viral infections is lost, whereas control over cytopathic viruses is more dependent on antibodies and interferon gamma (Kagi and Hengartner, 1996). Whether HIV-1 by itself is directly cytopathic for CD4 T-cells *in vivo* is unclear. This is an extremely important consideration, since lysis of infected cells is doubly important in HIV infection due to the ability of the virus to integrate and remain dormant in long-lived cells (Han et al., 2007). Therefore CD8 lysis of HIV-infected CD4 T-cells may be required to simultaneously reduce viral replication and proviral HIV DNA burden. Certainly, syncytium-inducing strains of HIV-1 are clearly directly cytopathic, but they are generally not found in early HIV-1 infection (Richman and Bozzette, 1994), so that CD8 control of non-syncytium-inducing strains during PHI is extremely important. In fact, it is widely believed that CD8 cytotoxicity is the main immune mechanism to control HIV infection (reviewed in detail in McMichael, 1998; McMichael et al., 2010).

Nevertheless, some early studies suggested that CD8 T-cells from LTNP had a non-cytotoxic, soluble factor-mediated suppressive activity, reducing HIV-1 replication *in vitro* (Walker et al., 1986; Yang et al., 1997; Barker et al., 1998; Wilkinson et al., 1999) as well as more recent studies suggesting significant suppression of HIV replication in autologous CD4 T-cells *in vitro* (Julg et al., 2010b). However, a lack of any mechanism elucidated for this suppression, particularly definition of any soluble mediator responsible (Levy, 2003) has seriously called this suppression into question. Furthermore, a recent study of EC clearly found that CD8 T-cells required contact with infected CD4 T-cells to reduce HIV replication, indicating cytotoxicity was the main mechanism (Saez-Cirion et al., 2007). Consistent with the role of cytotoxicity, an earlier study of HLA-B57 LTNP had shown that their HIV Gag-specific CD8 T-cells responded to antigen by proliferation and up-regulation of perforin (Migueles et al., 2002).

However, CD8 T-cells in LTNP may have more functions than just cytotoxicity. Betts et al. (2006) conducted a qualitative study to identify immunological correlates of disease protection, describing polyfunctionality as an important characteristic of CD8+ T-cell control that was inversely related to viral load in LTNP. Polychromatic flow cytometry was used to simultaneously assess five functional responses of antigen-specific CD8+ T-cells; production of TNFa, IL-2, IFNg, MIP1b, and up-regulation of CD107a (degranulation). LTNP displayed an enhanced functional profile of four or five simultaneous functions (Betts et al., 2006), also confirmed in HLA-B27 LTNP (Almeida et al., 2007, 2009).

Importantly, as for CD4 responses described above, a large study of HIV+ subjects in South Africa showed a clear association of lower viral loads with CD8 T-cell responses to Gag, while responses to Env were associated with higher viral loads (Kiepiela et al., 2007). It is presumed that a major reason is probably that escape mutations in Gag are highly constrained by their effect on fitness (Goulder and Watkins, 2004), as discussed above for HLA-B27 LTNP and their very gradual loss of control. In addition, it has been suggested that epitopes from Gag could be presented very early after viral entry into the target cell and before integration and productive infection (Sacha et al., 2007). As mentioned above, this could be doubly important in preventing establishment of the latent reservoir of HIV DNA infected long-lived memory CD4 T-cells, as observed in LTNP (Descours et al., 2012).

## CD8 TCR Functional Avidity and LTNP

Increased antigen sensitivity represents the efficiency of CD8+ T-cells to expand and respond to lower levels of antigens during infection; while potentially providing an advantage to controlling infection, this feature may also leave cells prone to exhaustion (Almeida et al., 2007; Lichterfeld et al., 2007). Also referred to as functional avidity, assessment is quantifiable (normally through EC_50_ of antigen titration), by measurement of function downstream of TCR signaling, such as proliferation and cytotoxicity. So, the question arises whether LTNP have CD8 T-cells that are highly sensitive to antigen and more effective at clearing infection.

Recruitment of T-cells with high avidity has been demonstrated to occur during early HIV infection and is associated with a decline in viremia (Lichterfeld et al., 2007). Study of CD8 T-cells from HLA-B27 HIV+ subjects showed that those individuals with CD8 T-cells that responded to lower antigen concentration, i.e., higher functional avidity, had the lowest HIV DNA in PBMC (Almeida et al., 2007) and plasma viral load (Almeida et al., 2009). High avidity CD8 T-cells were also much more effective in suppressing HIV-1 replication *in vitro* (Almeida et al., 2009; Bennett et al., 2010). However, some progressing HIV+ subjects may also have CD8 T-cells with apparent high avidity to autologous HIV-1, as measured by IFNg responses *in vitro* (Draenert et al., 2004), although polyfunctionality was not studied. Similarly, protective effects may also be associated with low to medium-avidity CD8 T-cells (Harari et al., 2007). Furthermore, high avidity CD8 T-cells appear to express higher levels of the dysfunction marker PD-1 (Harari et al., 2007), and the senescence marker CD57 (Almeida et al., 2007), and may be subject to higher rates of apoptosis (Vigano et al., 2012).

Therefore, it appears that high functional avidity CD8 T-cells *per se* do not necessarily lead to LTNP status, that other factors controlling longevity of these CD8 T-cells may also be important.

## CD8 Immunodominance and Clonal Turnover and LTNP

**Figure 2:**
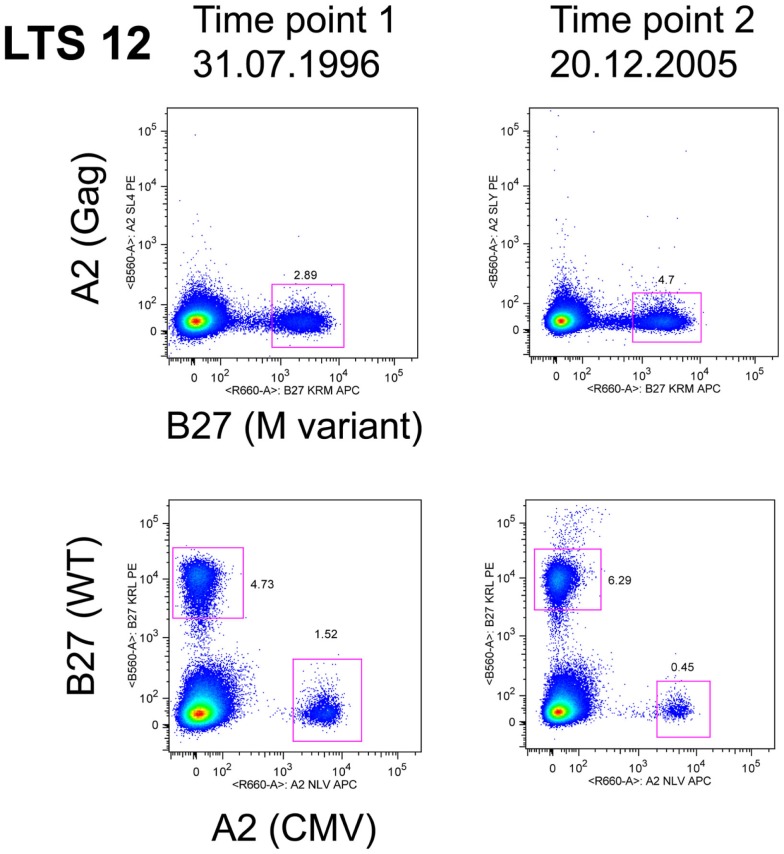
**LTS12 CD8 T cells binding to WT and L268M KK10 Tetramers**. Flow cytometry histograms showing that subject LTS12 had a very large CD8 T cell response to HLA-B27-restricted epitope KK10, both to the wild-type sequence peptide-tetramer (lower histograms) and to the L268M variant peptide-tetramer (upper histograms), over two time points separated by 9 years. Clonotyping of sorted tetramer+ cells showed that the dominant clonotypes were responding to both epitope sequences.

The evidence from HLA-B27 and HLA-B57 LTNP and EC suggests that efficient, immunodominant CD8+ T-cells responses maintain control over viral replication, but it is not known how this dominance is established, maintained, and linked with non-progression. Logically, cells that have the capacity to rapidly expand *in vivo* to limiting amounts of antigen have a competitive advantage and would predominate when confronted with the task of clearing pathogen. Recruitment of clonotypes making up the immunodominant response to the HLA-B*2705-restricted KK10 epitope is closely linked to viral replication. Treatment-naïve non-progressors recruited multi-functional KK10-specific CD8+ T-cells that were prone to replicative senescence following up-regulation of the marker CD57 (Almeida et al., 2007). However, a similar longitudinal study of the KK10 response noted the persistence of immunodominant clonotypes, which expressed markers of long-lived CD8 T-cells, namely high IL-7R and Bcl-2 (van Bockel et al., 2011). A notable feature of these long-lived immunodominant clones in the latter study was their ability to bind to tetramers containing the wild-type peptide, as well as to tetramers containing the mutant L268M peptide (van Bockel et al., 2011) as shown in Figure 2, and discussed below.

The factors controlling recruitment of clones and immunodominance are not well understood. New clonal responses can be recruited during chronic infection, a classic example being HLA-A2 individuals who do not have CD8 T-cell responses to the p17 peptide SLYNTVATL during PHI, but response to this peptide is immunodominant in 75% of HLA-A2 subjects with chronic infection (Goulder et al., 2001a).

Extended control over replication by effective HLA-B27-restricted CD8 T-cell clones may depend on their ability to respond to closely related variants as they arise. Altogether, there may be a complex dynamic of CD8 clonal recruitment in HLA-B27 subjects, followed by pressure exerted by these cells on HIV-1, resulting in appearance of variants in the epitope, which in turn affects the differentiation state and longevity of the antigen-specific CD8 T-cells.

## Cross-Reactive TCR may Prevent Effective Escape in HLA-B27

Considerable interest has recently focused upon cross-reactivity of T-cells for viral epitope variants. Given the very large numbers of possible peptide epitopes that could be presented by the MHC to T-cell receptors, it has been argued that a single TCR may recognize multiple epitopes (Mason, 1998). These theoretical considerations are supported by data in LTNP demonstrating TCR cross-reactivity by HLA-B*2705-restricted KK10 clones (Almeida et al., 2009; van Bockel et al., 2011; Chen et al., 2012) and HLA-B*5701/03-restricted clones (Gillespie et al., 2002; Turnbull et al., 2006; Simons et al., 2008) toward known viral variants. An example of an immunodominant clonotype that bound both wild-type and L268M KK10 peptides is shown in Figure 2, as originally described in van Bockel et al. (2011). A recent study has reported that KK10-specific immunodominant CD8 clonotypes from HLA-B27 LTNP are extremely effective at suppressing HIV-1 replication *in vitro*, with both wild-type KK10 and L268M viral strains suppressed, whereas HLA-B27 progressors had CD8 clonotypes that were clearly inferior at suppressing wild-type and did not suppress L268M strains at all (Chen et al., 2012). Suppression by CD8 T-cells from LTNP compared to progressors was most closely linked to rapid up-regulation of perforin (Chen et al., 2012).

One report suggested that protective alleles such as HLA-B57 may have less deletion of relevant TCR clonotypes during negative selection in the thymus, and therefore LTNP may have a greater number of potential clonotypes to select from during PHI (Kosmrlj et al., 2010). However, another report studied the diversity of HLA-B57-restricted anti-Gag clonotypes and found that LTNP and progressors had similar numbers and diversity of clonotypes (Mendoza et al., 2012b).

Therefore, the question of how LTNP have qualitatively superior CD8 T-cell responses remains unresolved, but their ability to simultaneously suppress HIV-1 replication by both wild-type and well characterized variants appears to be significant. Nevertheless, one report suggests that one HLA-B57 LTNP, infected originally with a *nef*-defective strain of HIV-1, was superinfected with a *nef*-intact HIV strain despite having detectable HLA-B57-restricted CD8 responses to the main HIV Gag epitopes (Braibant et al., 2010).

### NK cells and control of HIV-1 infection

NK cells typically represent 5–15% of circulating lymphocytes and may be one of the first mechanisms in the defense against HIV-1 infection, prior to development of adaptive immune responses. NK cells would be expected to lyse HIV-infected cells, since Nef downregulates HLA class I molecules to reduce presentation of antigens to cytotoxic CD8 T-cells (Collins et al., 1998), potentially triggering NK cell cytotoxicity due to “missing self”(Alter and Altfeld, 2009). However, Nef may selectively only downregulate HLA-A and -B, leaving HLA-C and -E expression intact, thereby also evading NK cells (Cohen et al., 1999). Loss of this function of Nef may be important in the reduced fitness of Nef-deleted virus in some LTNP, as described above. However, there are many other reported functions of Nef (Foster and Garcia, 2008) and it must be remembered that *nef* deletions overlap with 3′LTR (Gorry et al., 2007).

Nevertheless, genetic evidence suggests that HIV+ subjects with the activating KIR allele KIR3DS1, in combination with HLA-B alleles that encode molecules with isoleucine at position 80 (HLA-B Bw4-80Ile), have delayed progression to AIDS (Martin et al., 2002). It was subsequently found that this genetic combination resulted in NK cells from KIR3DS1+ subjects exhibiting higher levels of degranulation toward HIV-infected Bw4 80I CD4 target cells and suppression of HIV-1 replication (Alter et al., 2007).

Furthermore, a genetic polymorphism located 35 kb upstream of HLA-C has been found to be associated with slower HIV progression in genome-wide association studies (Fellay et al., 2007). It was later found that this polymorphism was in strong linkage disequilibrium with a variation within the 3′ untranslated region of HLA-C that regulates binding of the microRNA species miR148a resulting in relatively higher expression of HLA-C on the surface of cells (Kulkarni et al., 2011). Therefore higher levels of HLA-C, possibly related to NK cell activity, may be important for slow progression.

Recently, there has been renewed interest in another function of NK cells, namely antibody-dependent cell-mediated cytotoxicity (ADCC), in which NK cells with the Fc receptor CD16 lyse target cells that have antibody bound to antigens on their cell surface. ADCC was originally believed to be one mechanism contributing to the reduction in plasma viral load after PHI (Koup et al., 1994). Recently, ADCC responses to Env were correlated with slow decline in CD4 T-cell count (Chung et al., 2011).

## Humoral Responses to HIV

The antibody response to HIV-1 antigens begins around 1 week after onset of symptoms and is absolutely characteristic of PHI (Cooper et al., 1985; Tindall and Cooper, 1991). Antibodies to all components of HIV-1 gradually appear and intensify on western blot over several weeks (Tindall and Cooper, 1991). The observations that antibodies are predominantly class-switched IgG, that there is evidence of somatic mutation from germ-line Ig genes, that titers and affinity increase over time, and that there are numerous germinal centers within lymph nodes, all point to a significant T follicular helper (Tfh) CD4 cells-dependent antibody response to HIV-1 infection (Crotty, 2011).

Very early treatment of PHI results in decreasing titers of anti-p24 antibodies (Zaunders et al., 1999a) suggesting interruption of this slow build up of antibodies. In fact, it is speculated that decreasing antibody titers, which is rare even for EC subjects (Mendoza et al., 2012a), may actually reflect a complete lack of viral replication in tissues and decrease in available antigenic material in germinal centers. In contrast, in progressive infection, the amount of virions attached to the processes of follicular dendritic cells is overwhelming (Haase, 1999) and outnumber all other virions in the body. Altogether, these results are not consistent with the idea that HIV-specific CD4 T-cells are preferentially infected and deleted during HIV infection (Douek et al., 2002). Instead, very recent studies of Tfh in lymphoid tissue during SIV and HIV infection have shown that these cells increase during chronic infection, even in progressive disease (Hong et al., 2012; Lindqvist et al., 2012; Petrovas et al., 2012; Xu et al., 2013).

Therefore, the question arises whether LTNP and EC have a more effective neutralizing antibody response than HIV+ subjects with progressive infection. In studies of large numbers of subjects with slow disease progression, broadly neutralizing antibodies were uncommon, although some highly effective monoclonal antibodies have been generated from selected patients (Zhou et al., 2010; Walker et al., 2011). Overall, it is not believed that neutralizing antibodies play a major role in LTNP or EC status. However, it remains possible that ADCC, mediated by NK cells, may play a role in controlling HIV-1 replication in LTNP (Chung et al., 2011), as mentioned above.

## Conclusion

The predominant evidence suggests that LTNP and EC have effective CD4 and CD8 T-cell activities that in these rare cases are able to target critical epitopes in HIV-1 Gag and severely limit HIV-1 infection and replication. The distinguishing feature of these cells is their ability to rapidly proliferate, upregulate cytotoxic effector molecules, lyse infected cells, and also respond to typical variants that may arise.

While these insights have mainly come from LTNP and EC with HLA-B57 and HLA-B27, approximately half of LTNP/EC subjects do not have these alleles, and in some cohorts even lower proportions have these alleles (Saez-Cirion et al., 2013). Therefore, genome-wide genetic association studies were hoped to provide further clues to LTNP and EC status, but only confirmed known HLA associations (Pereyra et al., 2010).

With respect to CD4 and CD8 effector cells in LTNP/EC, it is still difficult to definitively separate cause and effect, since they may have characteristics of effector cells in a low viral load environment. The observation that such effector cells, especially large populations of proliferative Gag-specific CD4 T-cells, are not often seen in subjects who had progressive infection and have suppressive cART argues in favor of a causal relationship. A relatively low viral load environment during PHI for LTNP/EC may be the crucial difference.

How such responses can be generated therapeutically so that HIV+ subjects can avoid lifelong ART remains a significant challenge.

## Conflict of Interest Statement

The authors declare that the research was conducted in the absence of any commercial or financial relationships that could be construed as a potential conflict of interest.
